# Association between Respiratory Sleep Indices and Cardiovascular Disease in Sleep Apnea—A Community-Based Study in Cyprus

**DOI:** 10.3390/jcm9082475

**Published:** 2020-08-01

**Authors:** Frangiskos Frangopoulos, Ivi Nicolaou, Savvas Zannetos, Nicholas-Tiberio Economou, Tonia Adamide, Georgia Trakada

**Affiliations:** 1 Respiratory Department, Nicosia General Hospital, 215 Nicosia–Limassol Old Road, Strovolos 2029, Nicosia, Cyprus; frangopoulos@yahoo.com (F.F.); nikivi28@yahoo.com (I.N.); tadamide@mphs.moh.gov.cy (T.A.); 2 Department of Healthcare Management, Neapolis University, Pafos 8042, Cyprus; zannetos@gmail.com; 3 Division of Pulmonology, Department of Clinical Therapeutics, School of Medicine, National and Kapodistrian University of Athens, Alexandra Hospital, 11528 Athens, Greece; nt_economou@yahoo.it

**Keywords:** obstructive sleep apnea (OSA), respiratory event index (REI), oxygen desaturation index (ODI), hypertension, arrhythmias, heart failure, ischemic heart disease, stroke

## Abstract

Obstructive sleep apnea (OSA) is a chronic and prevalent disorder, strongly associated with cardiovascular disease (CVD). The apnea-hypopnea index (AHI), or respiratory event index (REI), and the oxygen desaturation index (ODI) are the clinical metrics of sleep apnea in terms of diagnosis and severity. However, AHI, or REI, does not quantify OSA-related hypoxemia and poorly predicts the consequences of sleep apnea in cardiometabolic diseases. Moreover, it is unclear whether ODI correlates with CVD in OSA. Our study aimed to examine the possible associations between respiratory sleep indices and CVD in OSA, in a non-clinic-based population in Cyprus. We screened 344 subjects of a stratified, total sample of 4118 eligible responders. All participants were adults (age 18+), residing in Cyprus. Each patient answered with a detailed clinical history in terms of CVD. A type III sleep test was performed on 282 subjects (81.97%). OSA (REI ≥ 15) was diagnosed in 92 patients (32.62%, Group A). REI < 15 was observed in the remaining 190 subjects (67.37%, Group B). In OSA group A, 40 individuals (43%) reported hypertension, 17 (18.5%) arrhythmias, 10 (11%) heart failure, 9 (9.8%) ischemic heart disease and 2 (2%) previous stroke, versus 46 (24%), 21 (11%), 7 (3.7%), 12 (6.3%) and 6 (3%), in Group B, respectively. Hypertension correlated with REI (*p* = 0.001), ODI (*p* = 0.003) and mean SaO_2_ (*p* < 0.001). Arrhythmias correlated with mean SaO_2_ (*p* = 0.001) and time spent under 90% oxygen saturation (*p* = 0.040). Heart failure correlated with REI (*p* = 0.043), especially in the supine position (0.036). No statistically significant correlations were observed between ischemic heart disease or stroke and REI, ODI and mean SaO_2_. The pathogenesis underlying CVD in OSA is variable. According to our data, hypertension correlated with REI, ODI and mean SaO_2_. Arrhythmias correlated only with hypoxemia (mean SaO_2_), whereas heart failure correlated only with REI, especially in the supine position.

## 1. Introduction

Obstructive sleep apnea (OSA) is a chronic condition characterized by the repetitive collapse of the upper airway during sleep, leading to oxygen desaturation, sympathetic activation and recurrent arousals [1]. OSA is a common disease. We recently found that the prevalence of intermediate to high risk for OSA is 50% in males and 18% in females in the general population of Cyprus [2].

Cumulative evidence has established OSA as an independent risk factor for hypertension, heart failure and stroke, cardiovascular and all-cause mortality [3,4]. Apneas/hypopneas and consequent respiratory arousals disrupt normal sleep and cause intermittent hypoxemia and reoxygenation, with or without transient hypercapnia, increased sympathetic and decreased parasympathetic activity and repetitive, large negative intrathoracic pressures [5,6]. Related consequences of these underlying pathophysiological events are oxidative stress, increased catecholamines and inflammatory state, which could be the underlying mechanisms of the vascular damage and cardiovascular disease (CVD) morbidity in OSA [6].

Currently, we use the apnea-hypopnea index (AHI) to diagnose OSA and quantify its severity. Previous studies demonstrated modest to moderate associations between AHI and CVD [3,7,8]. AHI is a simple metric count of the number of respiratory events per hour of sleep and it cannot describe all respiratory disturbances during sleep. The percentage of time during sleep with an oxygen saturation below 90% (TST90) predicts CVD more strongly than AHI [9]. However, TST90 characterizes both intermittent hypoxemia due to respiratory events and persistent hypoxemia due to other respiratory diseases (chronic obstructive pulmonary disease or interstitial lung disease).

The oxygen desaturation index (ODI) is the average number of desaturation episodes per hour of recording, as calculated by an algorithm—and not by manual scoring—based on information from a single channel (oximetry). Although ODI is commonly used in the assessment of OSA, its clinical significance and possible association with CVD is still not clear. In our study, we aimed to evaluate the possible correlations of ODI with CVD versus the respiratory event index (REI).

## 2. Subjects and Method

In an attempt to estimate the risk of sleep-disordered breathing in Cyprus, a telephone survey of a modified STOP-Bang questionnaire was conducted on a larger scale in the adult general population, as previously described [2]. The STOP-Bang questionnaire is a concise and easy to-use, self-reportable, screening tool for OSA that includes eight yes/no questions about snoring, tiredness, observed apnea, high blood pressure, BMI > 35 kg/m^2^, age > 50 years old, neck circumference > 40 cm, and male gender. In our survey, the question about neck circumference was removed from the final evaluation due to the uncertainty of most of the participants and the risk of bias. The initial participants included adult individuals, residing in Cyprus. The eligibility criteria for inclusion were as follows: (1) age ≥ 18 years old, (2) Cypriot citizens with permanent residence under the effective control of the Republic of Cyprus, and (3) consent and willingness to participate in the survey. The sample was stratified according to the last demographic report (2016) by district, rural or urban area, gender and age. Stratification ensures that the sample is representative of the population with respect to the chosen population parameters. The study was approved by the Cyprus Bioethics Committee (EEBK/EP/2016/35) and all subjects gave consent to participate in the study after appropriate information was given. The survey was conducted by the computer-aided telephone interviewing (CATI) method.

**Figure 1:**
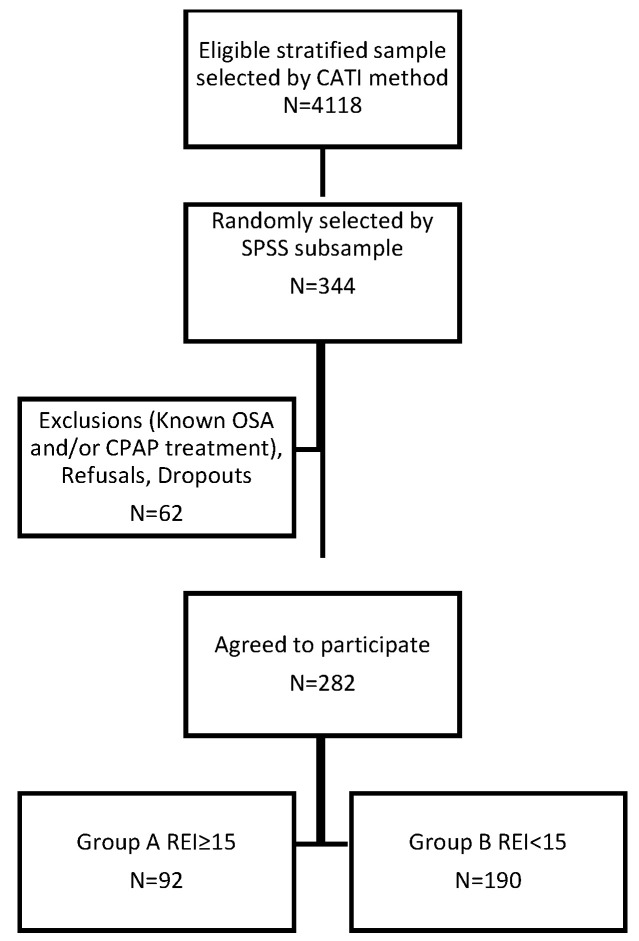
Study selection flow chart.

A secondary cross-sectional nationwide survey was piloted to examine the possible associations between the characteristics of sleep-disordered breathing measured by a type III sleep test and CVD in the adult population. A type III sleep testing device monitors a minimum of four channels that include one or more channels of respiratory effort, airflow, oxygen saturation, and heart rate/electrocardiogram. From the initial representative sample of the Cypriot adult population (4118 participants), 344 adults were randomly selected to participate in the second stage procedure (Figure 1). All subjects provided a detailed medical history about previously diagnosed CVD (hypertension, arrhythmias, heart failure, ischemic heart disease and previous stroke), diabetes mellitus, and chronic renal failure.

No strict inclusion or exclusion criteria were applied in order to guarantee no bias in the selection, minimize the necessary sample size and achieve reliable results. Participants were excluded from the analyses only if they had a previous known history of sleep apnea and were under treatment with continuous positive airways pressure (CPAP) or other therapies.

A type III sleep test was performed on 282 subjects (81.97%) and the following polysomnographic measures were used in the analyses: total recording time (TRT), respiratory event index (REI) (number of respiratory events per hour of recording), oxygen desaturation index (ODI) (number of ≥3% oxygen saturation drops per hour of recording), mean oxygen saturation during recording (mSaO_2_), and time spent under 90% oxygen saturation (TST90) (percentage of TRT spent under a 90% oxygen saturation threshold). Potential associations were examined in a double-blinded manner.

Statistical analysis included summarization of the data in tables and charts and it was performed by using a statistical analysis software platform (IBM SPSS Statistics v.25 program). Descriptive statistics procedures for survey data were used to examine characteristics for all participants. Hypothesis testing was performed in order to accept or reject the null hypothesis. Specifically, *χ*^2^ test was performed when comparing nominal variables, and *t* tests or ANOVA were performed when comparing continuous variables. All results reported are based on two-sided tests. When ANOVA tests were performed, all pairwise comparisons were checked using the Tukey post hoc test. A *p* value lower than 0.05 was considered significant.

## 3. Results

From 4118 eligible responders, stratified to represent the Cypriot population, a cohort of 344 individuals—randomly selected by SPSS—were enrolled and 282 subjects (81.97%) performed the type III sleep test.

Modified STOP-Bang questionnaire >3 was a sensitive screening index for REI > 5 and >15 and ODI > 15 (*p* < 0.001). OSA was diagnosed in 92 patients (32.62%). Men suffered more often than women (*p* = 0.007). Apneic patients were older and more obese (body mass index [BMI] > 35 kg/m^2^) compared to non-apneic (*p* < 0.001). TST90 was statistically significant and common in obese individuals (*p* = 0.047), but it did not differ between age groups >50 years and <50 years.

Among the total population, the prevalence of CVD was as follows: 30.49% for hypertension, 13.47% for arrhythmias, 6.02% for heart failure, 7.44% for ischemic heart disease and 2.83% for previous stroke. In sleep apneic patients the prevalence of CVD was 43%, 18.5%, 11%, 9.8% and 2%, respectively. Finally, the prevalence of OSA was 47% in hypertensive patients, 45% in patients with arrhythmias, 59% in patients with heart failure, 43% in patients with ischemic heart disease and 25% in patients with previous stroke.

**Figure 2:**
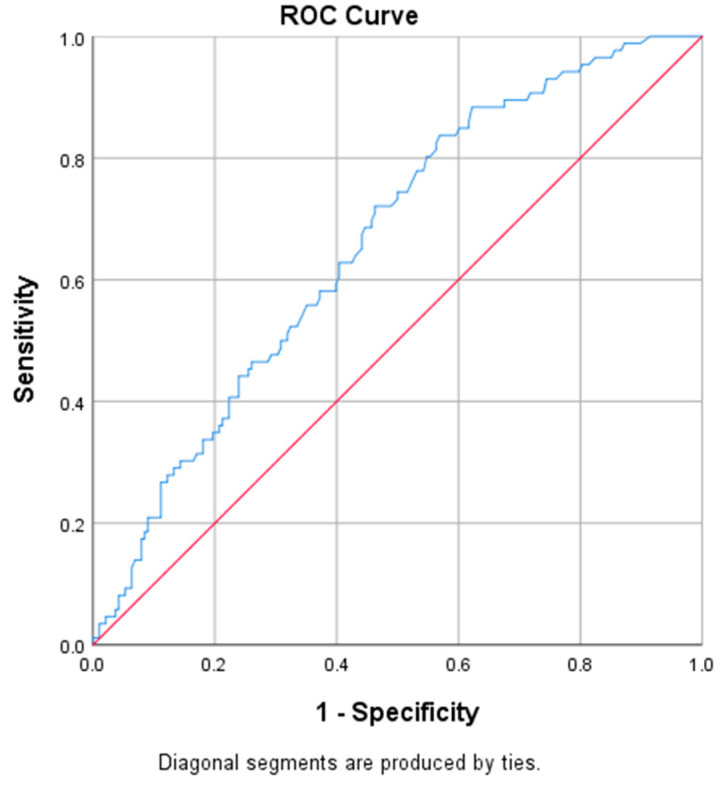
Hypertension: respiratory event index (REI) receiver operating characteristic (ROC) curve.

Hypertension was associated with all sleep study respiratory indices: REI > 5 (*p* < 0.001) and >15 (*p* = 0.004), ODI > 15 (*p* = 0.002), mSaO_2_ (*p* < 0.001) and TST90 (*p* = 0.019). A receiver operating characteristic curve, or ROC curve, is a graphical plot that illustrates the diagnostic ability of a binary classifier system as its discrimination threshold is varied. The ROC curve is created by plotting the true positive rate against the false positive rate at various threshold settings. The cut off value according to the ROC curve was 7.25 respiratory events per hour of recording (Figure 2). Hypertensive patients were older and more obese when compared to non-hypertensive—age: 63.81 ± 10.61 vs. 51.14 ± 12, 15 years, and BMI 30.54 ± 5.28 vs. 28 ± 5.13 kg/m^2^, respectively (*p* < 0.001).

Arrhythmias were associated with mean SaO_2_ (*p* = 0.001) and time spent under 90% oxygen saturation (TST90, *p* = 0.040). Patients with arrhythmias were older, with similar BMI, as compared to those without arrhythmias (age: 64.76 ± 10.47 vs. 54.30 ± 12.98 years, *p* < 0.001 and BMI: 29.90 ± 6.00 vs. 28.76 ± 5.22 kg/m^2^, *p* = 0.27, respectively) and suffered more frequently from hypertension (*p* < 0.001) and congestive heart failure (*p* = 0.016).

**Figure 3:**
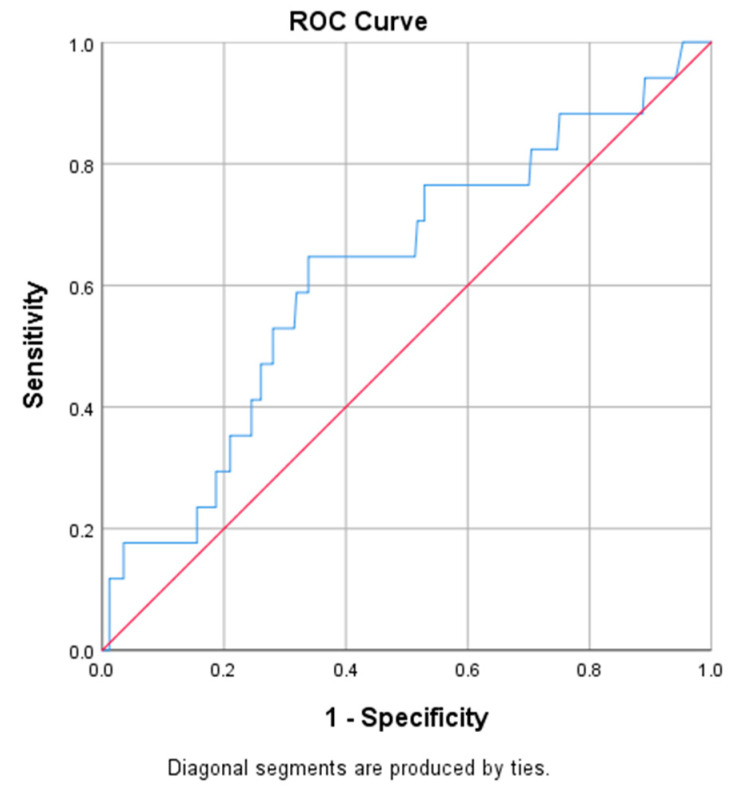
Heart Failure: REI ROC curve.

Heart failure (HF) was associated with REI (*p* = 0.043), especially in the supine position (0.036). The cut off value was 14.25 respiratory events per hour of recording (Figure 3). Patients with heart failure were also older, with similar BMI, as compared to those without HF (age: 62.82 ± 8.17 vs. 55.28 ± 13.29 years, *p* = 0.002 and BMI: 29.23 ± 5.94 vs. 28.90 ± 5.30 kg/m^2^, *p* = 0.824, respectively) and suffered more frequently from hypertension (*p* = 0.001), arrhythmias (*p* = 0.012) and ischemic heart disease (*p* = 0.004).

DeLong’s test for the comparison of the two ROC curves was used using the R language for statistical computing. There were no statistical differences between REI predictability for hypertension and heart failure (DeLong’s test for two ROC curves) [10].

No statistically significant correlations were observed between ischemic heart disease or stroke and REI, ODI and mean SaO_2_ or TST90.

## 4. Discussion

An epidemiologic community-based cohort study of 344 subjects aged 18–83 years was conducted in Cyprus and subjects were divided in two groups according to REI< or ≥15. The two groups were similar in age, gender and BMI. Sleep study indices, namely respiratory events index (REI), oxygen desaturation index (ODI) and mean oxygen saturation (meanSaO_2_) correlated to the cardiovascular history reported by the subjects. Hypertension correlated with REI, ODI and mean SaO_2_. Arrhythmias correlated only with hypoxemia (mean SaO_2_), whereas heart failure correlated only with REI, especially in the supine position.

Screening a non-clinic-based population identified many individuals with OSA as expected according to the prevalence [2]. It is possible that OSA in such a sample carries a lower cardiovascular risk than OSA in individuals presenting for evaluation in a sleep laboratory, but CVD remains the main cause of morbidity and mortality in the adult population and history reports from the individuals were revealing.

Obstructive Sleep Apnea (OSA) syndrome is an independent risk factor for cardiovascular morbidity and mortality. It is characterized by intermittent recurrent pauses in respiration. The mechanisms by which OSA may cause CVD are complex and include mechanical, neural, humoral and circadian rhythm components. Hypoxic and oxidative stress resulting from repeated episodes of hypoxemia and reoxygenation [11] cause systemic inflammation, endothelial dysfunction, increased production of vasoactive substances, insulin resistance and sympathetic nervous system activation, favoring triggered atrial and ventricular activity and abnormal automaticity [12]. It is hypothesized that these result in vascular dysfunction, hypertension, hyperlipidemia, diabetes and arrhythmias mediating the cardiovascular consequences of OSA.

Not all patients develop cardiovascular complications and possibility resides in the existence of large, individual differences in the response to OSA’s pathophysiological consequences and an individual’s susceptibility to CVD. Such discrepancies were observed in models of intermittent hypoxia and OSAS [13,14] and can be connected with the degree of reactive oxygen species (ROS, are chemically reactive species containing oxygen) production and/or decreased antioxidant capacity and subsequent inflammatory and adaptive pathways [15]. Therefore, it is likely that the genetic variations of patients with OSAS will determine the sensitivity to nocturnal hypoxia, autonomous system deregulation and mechanical strain. However, several factors, such as the depth, the duration and the intermittency of the hypoxia, dependent on OSA severity and complexity, are also crucial in the development of CVD.

OSA is recognized as an independent risk factor and a preventable cause of hypertension [16]. Hypertension is a risk factor for arrhythmias and heart failure, and this association makes it difficult to prove a causal relation between OSA and aggregated comorbidities like heart failure independent of arterial hypertension in epidemiological studies. Our results—hypertension correlated with REI, ODI and mean SaO_2_—support the hypothesis that all pathways represented by the above indices, namely mechanics (REI), intermittent hypoxia and autonomous system deregulation (ODI) and hypoxia/hypercapnia (mean SaO_2_), contribute to increased blood pressure. Interestingly, TST90 had no significant importance in our study, implying that time spent under the conventional 90% blood oxygen saturation is not a key point in the pathophysiology of CVD. Other studies have shown that individual obstruction events, with emphasis on a longer duration and a more severe oxygen desaturation during the events, increase blood pressure and mortality [17,18,19] independently from AHI severity. Additionally, novel OSA treatments benefit patients by decreasing events severity rather than AHI [20].

OSA gives rise to sympathetic activation, increased blood pressure and oxidative stress coagulopathy and therefore is considered a risk factor for the development of coronary disease, ischemic heart disease, hypertension cardiopathy and finally, heart failure. In our study, REI was the defining sleep study factor correlated with heart failure in accordance with other studies [4]. Heart failure risk was associated with REI ≥ 15 and was nominally statistically significant. Several studies have documented a high prevalence of OSA in patients with systolic and diastolic heart failure. On the other hand, heart failure causes ventilatory control instability and, through periodic reduction in neural output to both the diaphragm and pharyngeal dilator muscles, predisposition to sleep apnea. The present study demonstrates that individuals with OSA have higher risk of heart failure than persons without OSA. This may reflect the pathophysiologic importance of large intrathoracic pressure changes resulting from respiratory efforts against a collapsed upper airway, with consequent increases in left ventricular transmural pressure. Studies have suggested that negative intrathoracic pressure swings contribute to left ventricular hypertrophy and impaired cardiac function [21]. Transversely, these detrimental mechanical effects are more pronounced in those with chronic heart failure [22]. Our findings—heart failure correlated only with REI, especially in the supine position—strongly suggest that the mechanical encumbrance can be the critical determinant of HF and OSA interaction. The supine position is known for the deterioration of obstructive respiratory events and trepopnea is a key feature in the clinical presentation of heart failure.

Causality between OSA and arrhythmia has yet to be established. A range of mechanistic, complex and synergistic pathways may increase the propensity of cardiac arrhythmogenesis in OSA. Immediate pathophysiologic pathways include intermittent hypoxia, intrathoracic pressure swings and hypercapnia. Influences of OSA-enhancing arrhythmogenesis include increased systemic inflammation, oxidative stress, enhanced prothrombotic state, vascular dysfunction and autonomic nervous system fluctuations (enhanced parasympathetic activation during respiratory events and sympathetic activation subsequent to respiratory events). Comorbidities associated with OSA such as hypertension, heart failure and coronary artery disease also contribute to augmented arrhythmic propensity [23]. Our findings—arrhythmias correlated only with hypoxemia (mean SaO_2_)—suggest that OSA-related arrhythmias are mainly attributed to hypoxemia and probably the subsequent hypercapnia. Both lead to enhanced arrhythmia susceptibility [24]. Obstructive respiratory events impair gas exchange and lead to oxygen desaturation, particularly in individuals with underlying pulmonary or cardiovascular disease. Hypoxemia directly stimulates chemoreceptors in the carotid body, precipitating increased ventilation and sympathetic discharges. In addition, hypoxia leads to peripheral vasoconstriction, which increases both preload and afterload, thereby causing increased cardiac workload and strain. Reoxygenation may lead to the formation of hazardous reactive oxygen species and detrimental oxidative stress linked to arrhythmogenesis [25].

Our study has a number of advantages. The community-based, randomly selected sample identified a sleep lab-naïve sample that is optimal for the study. As subjects were recruited from the community rather than the clinic, there is no referral bias causing a spurious association of OSA with risk of cardiovascular disease and as OSA patients were excluded from the study, a better assessment of the natural history of untreated OSA is possible. Limitations must also be acknowledged. A thorough collection of detailed data concerning the history of cardiovascular disease was assembled but no cardiological assessment was provided in those with a negative CVD history in order to identify asymptomatic subjects. Unmeasured cardiovascular risk factors, such as diet, exercise and smoking habits, were not assessed and confounding by these variables cannot be excluded. Sex differences were not assessed in the study due to the confounding sample size, which included only 36% women. There are a number of possible explanations for the observed sex difference in the attendance of the study. The low prevalence of severe OSA in women is a limiting cause for volunteering. Another possible explanation is the later age of onset of OSA in women than in men, and finally, sex differences in the prevalence of cardiovascular disease may reflect a protective effect of female sex against cardiovascular risk, including risk related to OSA. Despite these limitations, the current study provides evidence that OSA indices are independently connected to certain CVDs, therefore suggesting that pathophysiological mechanisms contribute unequally in establishing different CVDs. Further studies examining other potential sleep disturbance variables, such as the arousals and the mean duration of obstructive events (the last may be highly predictive of mean oxygen saturation and TST90), and hypercapnia (as a potential arrhythmogenic factor), should be designed.
